# Stroke Secondary to Iron Deficiency Anemia: A Case Report

**DOI:** 10.7759/cureus.19526

**Published:** 2021-11-13

**Authors:** Natalie N Kandinata, Logen Breehl, Bhaskar Chhetri, Suresh Paudel

**Affiliations:** 1 Internal Medicine, Cape Fear Valley Medical Center, Fayetteville, USA

**Keywords:** stroke, iron deficiency, anemia, risk factors, cerebrovascular accident

## Abstract

Cerebrovascular accident is the fifth leading cause of death in the United States, with about 795,000 cases reported to the Centers for Disease Control and Prevention (CDC) each year. Several risk calculators for the development of stroke have been developed throughout the years, but none included iron deficiency anemia (IDA). We therefore would like to highlight the case of a 34-year-old female with severe iron deficiency anemia secondary to menorrhagia who had an ischemic stroke. An extensive workup was done and was negative. Given its significant presence with other comorbidities and various proposed pathogenesis, we propose that iron deficiency anemia be considered as a stroke factor. Studies in optimal hemoglobin or iron levels in patients with stroke to lower comorbidities and predict prognosis may also be beneficial.

## Introduction

Cerebrovascular accident is the fifth leading cause of death in the United States, with about 795,000 cases reported to the Centers for Disease Control and Prevention (CDC) each year [1]. Several risk calculators for stroke have been developed throughout the years, but none included iron deficiency anemia (IDA). The pathophysiology of strokes from anemia is interesting, as there are several proposed mechanisms for it. The spectrum of numerous anemia leads to stroke in different ways. First, anemia causes a hyperdynamic state that disrupts the integrity of the endothelial lining, which not only increases the risk of thrombus formation as per Virchow's triad but also dislodges the clot-producing arterial embolic strokes. The endothelial dysfunction itself causes further inflammation, leading to ischemic brain tissue damage in the setting of anemic hypoxia, especially in the watershed region of the brain. Second, erythropoietin levels are elevated in anemia, especially iron deficiency anemia (IDA). Its molecular pattern is similar to thrombopoietin, causing it to interact with receptors at the surface of megakaryocytes and eventually resulting in the proliferation of megakaryocytes and the production of mature platelets causing secondary reactive thrombocytosis. This could lead to venous sinus thrombosis or central retinal thrombosis. An arterial embolus can also happen. Third, altered deformability of red blood cells impairs oxygen delivery to tissues [2,3].

The purpose of this article is to report an interesting case of ischemic stroke in a young woman with iron deficiency anemia secondary to menorrhagia who otherwise had minimal risk factors. Given this case, we would like to propose that anemia be considered a stroke risk factor.

## Case presentation

A 34-year-old female with no significant past medical history other than tobacco smoking presented to the emergency department after she began to have trouble speaking and right arm weakness that started the day before. She denied significant alcohol use or any illicit drug use. She had no family history of hypercoagulability, blood disorders, or stroke.

Initial physical examination showed a right facial droop without ocular involvement. She had a focal strength deficit of her right wrist and hand, with hyperactive right brachioradialis tendon reflex. She was alert and oriented x3. All other neurologic examinations were within normal limits.

Laboratory analysis was significant for a hemoglobin of 9.1 g/dL, a hematocrit of 31.3%, an MCH of 19.6 pg, and an MCV of 66.9 fL.

**Figure 1:**
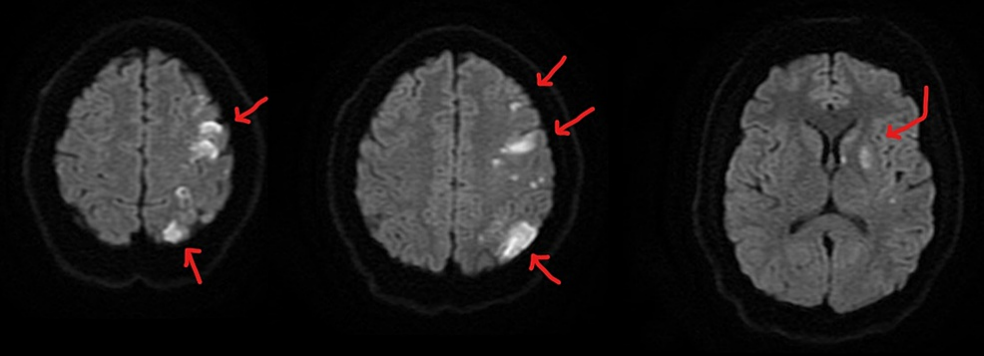
Patient's head MRI revealed multifocal acute infarcts – largest in the left parietal lobe measuring 2.7 × 1.4 cm and the left frontoparietal region measuring 2.6 × 1.6 cm, besides multiple smaller infarcts in the left basal ganglia, genu of the left internal capsule, and left frontal and parietal lobes.

Computed tomography (CT) of the head without contrast showed a hypodensity within the left frontal and parietal lobes. Computed tomography angiography (CTA) of the head showed no abnormal stenosis or occlusion. Magnetic resonance imaging (MRI) of the head, as seen in Figure 1, revealed multifocal infarcts.

Transthoracic echocardiogram (TTE) with bubble study showed an ejection fraction greater than 55%, mildly enlarged left atrium, trace mitral regurgitation, mild-moderate tricuspid regurgitation, and no patent foramen ovale (PFO). Transesophageal echocardiogram (TEE) showed no left atrial appendage thrombus, PFO, or atrial septal defect (ASD). Electroencephalogram (EEG) testing showed normal awake and sleep data and was otherwise unremarkable.

Hypercoagulability workup included diluted Russell viper venom time (dRVVT), prothrombin mutation, factor V level, lupus anticoagulant, protein C, and protein S free and total. Protein S total and free levels were slightly decreased at 57% and 46%, respectively. Autoimmune workup, including antichromatin antibody; cardiolipin immunoglobulin A (IgA), immunoglobulin G (IgG), and immunoglobulin M (IgM); anti-ribonuclear protein (RNP) antibodies, anti-Smith (anti-Sm) antibodies, anti-double-stranded deoxyribonucleic acid (anti-dsDNA), anti-scleroderma-70 (anti-Scl 70) antibody, Sjogren's antibodies A and B (anti-SSA and anti-SSB, respectively), anti-Jo-1, anti-centromere, and rheumatoid factor antibodies, was also completed, which were all negative. Prothrombin time (PT), partial prothrombin time (PTT), international normalized ratio (INR), glycated hemoglobin (HbA1c), lipid panel, and thyroid studies were also performed and found to be within normal limits. Urine toxicology screen was negative. There were no arrhythmias noted on telemetry throughout her five-day hospital course.

Anemia studies were significant for iron of 10 ug/dL, ferritin of 3.2 ng/mL, 3% iron saturation, and total iron-binding capacity (TIBC) of 338 ug/dL. Erythrocyte sedimentation rate and C-reactive protein were unknown.

On further history, it was found that she has been having issues with menorrhagia. The patient’s hemoglobin continued to drop from 9.1 g/dL on admission to 8.6 g/dL and then to 8.3 g/dL. She received multiple doses of intravenous iron during admission for treatment, and her condition continued to improve every day. Her hospital course was unremarkable, except for one episode of hypotension that resolved itself.

Although one could argue that she could have protein S deficiency causing stroke, her decreased levels were not significant. It likely decreased due to an acute phase reaction, as studies have shown that there is a very rare incidence of arterial stroke secondary to protein S deficiency, especially in the younger patient population under 45 years of age [4,5].

Due to the lack of other explanation for her multifocal acute infarcts, we conclude that this embolic stroke of undetermined cause (ESUS) was likely actually caused by iron deficiency anemia secondary to menorrhagia, as she was undergoing menses at the time she presented.

## Discussion

Cerebrovascular accident is the fifth leading cause of death in the United States, with about 795,000 cases reported to the CDC each year [1]. There are two types of strokes: hemorrhagic and ischemic. Hemorrhagic strokes generally occur within the parenchyma or subarachnoid space of the brain. Ischemic strokes are more common, making up about 80% of cerebrovascular accidents in the United States. It can be further broken down to cardioembolic, atherosclerotic, lacunar, others (such as dissection, vasculitis, and genetic disposition), or idiopathic [6]. Almost 25% of stroke cases are in patients under 65 years old, and 30% of them are due to undeterminable etiology [6]. Other causes of stroke include sudden hypotension, cerebral venous sinus thrombosis, cocaine use, different types of anemia, and idiopathic. Of patients with sickle cell disease, 24% have had at least one episode of a stroke by the age of 45, with ischemia-reperfusion injury being the most common mechanism. In thalassemias, the precipitation of red blood cells makes them nonfunctional, making the patient anemic and placing them at high risk of thromboembolic events. Overt strokes occur in 28% of patients with thalassemia major. Several studies reported a correlation between iron deficiency anemia and stroke in children [6]. Hartfield et al. reported an ischemic stroke or venous thrombosis in children with IDA post viral prodrome [7], while Azab et al. found that children with stroke are more likely to develop iron deficiency anemia post stroke [8]. There was a significant correlation between iron deficiency anemia and thrombocytosis found in the study. Regardless of the cause or effect, there is an undeniable correlation between iron deficiency anemia and stroke [3].

Certain stroke risk factors, such as age, gender, genetics, and race/ethnicity, are unmodifiable, but many others are. Different scales, such as Framingham and INTERSTROKE, have been used to estimate risk factors, and it is the healthcare provider’s duty to understand what they are and work with patients to maximize health and minimize these risks. The Framingham stroke risk factors include age, sex, systolic blood pressure, antihypertensive medication use, diabetes, smoking, atrial fibrillation, left ventricular hypertrophy, and prevalent coronary heart disease. The INTERSTROKE study in 22 countries determined modifiable risk factors to be hypertension, current tobacco use, waist/hip ratio, diet risk score, regular PA, diabetes, high alcohol consumption, stress/depression, cardiac causes, and apolipoprotein B (ApoB)/apolipoprotein A (ApoA) ratio. Out of all these risk factors, hypertension especially plays a huge role in hemorrhagic strokes, especially in developing countries. Blood pressure is affected not only by diet and exercise but also by other medical conditions and increases with age, which is not a modifiable risk factor. As one can see, iron deficiency anemia is such an important risk factor to control as it is often a manifestation of poor health. Similar to hypertension, anemia is often present in patients with chronic illnesses and multiple comorbidities, and it would be reasonable to consider it a risk factor for stroke [3].

## Conclusions

In the light of this case and several similar case reports, we think iron deficiency anemia should be considered as a possible risk factor for stroke, and more aggressive screening and treatment for IDA should be considered. Further studies need to be done as well to see if iron deficiency anemia can be a prognosis factor in patients with stroke.
